# Identification of Halophilic Microbes in Lung Fibrotic Tissue by Oligotyping

**DOI:** 10.3389/fmicb.2018.01892

**Published:** 2018-08-30

**Authors:** Corina N. D’Alessandro-Gabazza, Celia Méndez-García, Osamu Hataji, Sara Westergaard, Fumiaki Watanabe, Taro Yasuma, Masaaki Toda, Hajime Fujimoto, Kota Nishihama, Kentaro Fujiwara, Osamu Taguchi, Tetsu Kobayashi, Roderick I. Mackie, Isaac Cann, Esteban C. Gabazza

**Affiliations:** ^1^Department of Immunology, Mie University, Tsu, Japan; ^2^Carl R. Woese Institute for Genomic Biology, University of Illinois at Urbana–Champaign, Urbana, IL, United States; ^3^Department of Animal Sciences, University of Illinois at Urbana–Champaign, Urbana, IL, United States; ^4^Respiratory Center, Matsusaka Municipal Hospital, Matsusaka, Japan; ^5^Department of Diabetes and Endocrinology, Mie University, Tsu, Japan; ^6^Department of Pulmonary and Critical Care Medicine, Mie University, Tsu, Japan; ^7^Center for Physical and Mental Health, Mie University, Tsu, Japan; ^8^Department of Microbiology, University of Illinois at Urbana–Champaign, Urbana, IL, United States

**Keywords:** fibrosis, microbes, epithelial cells, cancer, mouse model, lung tissue

## Abstract

Idiopathic pulmonary fibrosis (IPF) is an incurable disease with poor prognosis and unknown etiology. The poor clinical outcome is associated with enhanced microbial burden in bronchoalveolar lavage fluid from IPF patients. However, whether microbes from the respiratory tract fluid cause the disease remains uncertain. Tissue-associated microbes can influence host physiology in health and disease development. The aim of this study was to evaluate the existence of microbes in lung fibrotic tissues. We evaluated the microbial community in lung tissues from IPF and from human transforming growth factor-β1 (TGF-β1) transgenic mice with lung fibrosis by oligotyping. We also evaluated the microbial population in non-tumor-bearing tissues from surgical specimens of lung cancer patients. The phyla *Firmicutes* and the genus *Clostridium* tended to be predominant in the lung tissue from IPF and lung cancer patients. Oligotyping analysis revealed a predominance of bacteria belonging to the genera *Halomonas, Shewanella, Christensenella*, and *Clostridium* in lung tissue from IPF and lung cancer. Evaluation of the microbial community in the lung tissue from mice revealed abundance of *Proteobacteria* in both wild-type (WT) littermates and transgenic mice. However, the genus *Halomonas* tended to be more abundant in TGF-β1 transgenic mice compared to WT mice. In conclusion, this study describes tissue-associated microbes in lung fibrotic tissues from IPF patients and from aging TGF-β1 transgenic mice.

## Introduction

Idiopathic pulmonary fibrosis (IPF) is a progressive and fatal disease with a median survival of only 2–4 years following diagnosis, and thus it is considered to be more lethal than many types of cancer (King et al., 2011). No definite etiology of IPF has been identified and no effective therapy that shows survival benefit is available (King et al., 2011). It is currently thought that the disease is the result of a chronic/repetitive injury and dysfunction of alveolar epithelial cells triggered by environmental or occupational factors (e.g., pollutants, dusts, gastric aspirates, and cigarette smoke) in a genetically predisposed host (Richeldi et al., 2017). Epithelial injury is apparently followed by increased expression of pro-fibrotic cytokines and growth factors, accelerated apoptosis of the alveolar epithelium, abnormal accumulation of activated myofibroblasts, and excessive deposition of extracellular matrix proteins in the lungs (Richeldi et al., 2017). The mechanism of the lung epithelial dysfunction induced by the gene and environmental risk factors is unclear. Recent studies suggest that imbalance of the lung microbial community or dysbiosis may play a role in the process of chronic injury and activation of lung epithelial cells in IPF (Molyneaux and Maher, 2013; Hewitt and Molyneaux, 2017). The association of the lung microbiota with acute exacerbation, clinical progression of the disease, risk of death, decline in pulmonary function, persistent elevation of the host immune response and with the progression-free survival, and fibroblast responsiveness provides strong evidence on the clinical and pathogenic relevance of the lung microbiota in IPF (Molyneaux et al., 2014; Huang et al., 2017; Wang et al., 2017). However, whether the lung microbial population is the cause or consequence of IPF remains unknown.

Studies on the gut microbiome have shown that mucosa-associated microbes are relatively stable overtime and that bacteria directly interacting with organ tissue can affect host response in health and disease development (Backhed et al., 2005). Most studies on the microbiome in IPF have used bronchoalveolar lavage fluid (BALF) samples for microbial analysis (Han et al., 2014; Molyneaux et al., 2014); that is, most isolated microbes were residing in the airway lumen. Another limitation of past studies is the use of methods with restricted taxonomic resolution, and therefore only microorganisms commonly found in healthy individuals could be detected in the IPF lung (Han et al., 2014; Molyneaux et al., 2014; Kitsios et al., 2018). In this study, we hypothesized that the use of a high-resolution computational method would allow identification of microbial communities previously undescribed in the lung microbiome. To test this hypothesis, we prepared and sequenced 16S ribosomal DNA amplicons from lung tissue samples and used oligotyping, a computational method with high resolution (Eren et al., 2013, 2014) to distinguish microbial communities in fibrotic tissues from patients with IPF and transforming growth factor (TGF)-β1 transgenic mice with pulmonary fibrosis.

## Materials and Methods

### Subjects

This study comprised six patients with IPF, three patients with lung adenocarcinoma, two patients with collagen vascular disease-associated interstitial lung disease, one subject with pneumothorax, and three healthy subjects (**Supplementary Table 1** and **Supplementary Figure 1**). Diagnosis of IPF and lung cancer was done following accepted international criteria (Travis et al., 2011; Meyer et al., 2012).

All subjects provided written informed consent and the study protocol was approved by the Ethical Committees for Clinical Investigation of Mie University (Approval No. 2707, March 7, 2014), Matsusaka Municipal Hospital (approval dated June 11, 2014), and Mie Chuo Medical Center (Approval No. 2014-6; September 2, 2014) and conducted following the principles of the Declaration of Helsinki.

### Sample Collection From All Subjects

None of the subjects included in the study received any therapy with antibiotics 6 weeks prior to sample collection. Bronchoscopy study was performed following guidelines of the American Thoracic Society and BALF samples were collected as previously described (Meyer et al., 2012; Han et al., 2014). Aliquots of unprocessed BALF collected into sterile tubes were immediately placed on ice, centrifuged at 15,000 rpm at 4°C for 30 min and each pellet was frozen at -80°C until DNA extraction. Unstimulated saliva was collected from all study participants after thorough flushing with a water rinse. The subjects were instructed to expel continuously spit that pools on the floor of the mouth on sterile plastic tubes, transferred to sterile tubes, centrifuged as described above and the pellet stored at -80°C until DNA extraction. Histopathological diagnosis of IPF was done in five patients through lung samples obtained by video-assisted thoracoscopic surgery (VATS), which was performed following standard sterile surgical conditions (Morris and Zamvar, 2014). Lung samples obtained by VATS from three IPF patients were available for microbial analysis. Lung histological samples from lung cancer patients were obtained from the resected lung tumor after completion of the therapeutic surgical procedures (**Supplementary Table 1**). The normal non-tumoral regions of the resected lobe from lung cancer patients were used for the preparation of bacterial genomic DNA.

### Animals

Human TGF-β1 transgenic mice backcrossed to C57BL/6J mice for more than 10 generations were previously characterized and wild-type (WT) littermates were used as controls (D’Alessandro-Gabazza et al., 2012). All animals were maintained in a specific pathogen-free environment and subjected to a 12-h light/dark cycle in the animal house of Mie University. Micro-computed tomography (micro-CT) of mice was performed as previously described (D’Alessandro-Gabazza et al., 2012). The Committee for Animal Investigation of Mie University approved the experimental protocols (Approval No. 24-50) and all procedures were performed in accordance with approved institutional guidelines.

### Experimental Design and Sample Collection From Mice

Transforming growth factor-β1 transgenic mice (females, *n* = 7, males, *n* = 5) of more than 20 weeks of age and with advanced pulmonary fibrosis on micro-CT examination were used in the experiments. WT (females, *n* = 5; males, *n* = 2) littermates with normal findings on micro-CT examination were used as controls. For microbial analysis of lung tissue, mice were euthanized by an overdose (>120 mg/kg) of sodium pentobarbital administered by intraperitoneal injection and the lungs were sampled using sterilized instruments and under sterile surgical conditions. Excised lung specimens were placed into sterile tubes and stored at -80°C until analysis.

### DNA Isolation From Samples

To extract DNA from BALF and saliva, the samples were centrifuged at 12,000 × *g* for 10 min at 4°C, the pellets were suspended in a total of 100 μl saline and transferred to 2-ml bead beating tubes (Mo Bio). DNA was extracted using the Soil DNA Isolation Kit, Power Soil (Mo Bio) according to the manufacturer’s instruction. DNA concentration was quantified using the NanoDrop^®^ ND-1000 Spectrophotometer (Nanodrop Technologies). Frozen lung tissues were placed in a sterile plastic dish, dissociated, cut and finely minced using sterile scalpels and then DNA was extracted following the same procedure described above and prepared for subsequent analysis.

### Small Subunit Ribosomal Ribonucleic Acid (SSU rRNA) Hypervariable Tag Sequencing

Amplicon libraries for the 16S rRNA gene V1–V3 hypervariable regions were constructed using a set of primers targeting the hypervariable regions V1–V3 (V123), using a mixture of four forward primers V123F1234, and one reverse primer V123R, as previously described (Trompette et al., 2014). The products obtained from six separate reactions were pooled and purified using Gel Band Purification Columns (GE Healthcare, United Kingdom), and the DNA concentration was determined using the Qubit^®^ dsDNA HS Assay Kit (Life Technologies, United States). Sample-specific, 12-base barcodes were used to tag each PCR product prior to high-throughput sequencing. The uniquely tagged, pooled DNA samples were immobilized on DNA capture beads, amplified through emulsion-based clonal amplification (emPCR), and sequenced in a 454 Life Sciences Genome Sequencer FLX (Roche Diagnostics) at the W.M. Keck Center for Comparative and Functional Genomics, University of Illinois at Urbana–Champaign and at the Microarray Technologies and BIOInfOSU Core Facility Services at Oklahoma State University, Stillwater. There are reports showing comparable results obtained using either 454 or the now broadly used Illumina MiSeq for the scrutiny of microbial biodiversity through 16S rRNA gene sequencing (Tremblay et al., 2015; Castelino et al., 2017).

### Community Analysis

514,985 quality-filtered 16S rRNA sequences from 454 pyro-sequencing were processed using the QIIME pipeline (Caporaso et al., 2010). Reads with lengths below 200 nucleotides and quality scores under 25 were excluded from further analysis. No mismatches were allowed in the forward primer. Resulting quality-controlled sequences were denoised using default settings and binned into operational taxonomic units (OTUs) at a 97% sequence similarity cutoff using uclust 1.2.22 as OTU picking method (Edgar, 2010; Kunin et al., 2010). The cluster seed was used as representative sequence. Chimeric sequences were detected with the Chimera Slayer algorithm and excluded from subsequent analysis. Non-chimeric sequences were aligned with the PyNAST tool using as reference the Greengenes core set alignment (DeSantis et al., 2006; Caporaso et al., 2010). Taxonomy assignations were inferred through comparisons with both the RDP and BLASTn databases (Altschul et al., 1990; Cole et al., 2009). Rarefaction analysis was performed at a depth of 1000 sequences by sample in order to remove the heterogeneity of the number of sequences by sample prior to calculation of alpha and beta diversity statistics. Alpha diversity was measured through Shannon (measurement of the diversity), Chao1 (richness), and equitability (evenness) indices. Beta diversity was calculated using weighted and unweighted UniFrac metrics. QIIME data were imported to the R software for further statistical inference (R Core Team, 2015). Barplots and boxplots were created with ggplot2 after exclusion of taxa with relative abundances under 1% (Wickham, 2009).

### Staining of Proliferating Cell Nuclear Antigen

Staining of PCNA was performed at Biopathology Institute Corporation (Oita, Japan) using rabbit polyclonal anti-PCNA antibody (Abcam, Tokyo, Japan) and biotin-labeled anti-rabbit IgG antibody according to standard methods.

### Oligotyping Analysis

QIIME data were used as input for the oligotyping pipeline in order to determine oligotypes within the taxa contributing to variation among samples (Eren et al., 2013). Each oligotype was required to appear in at least one sample (-s 1) and have a minimum substantive abundance of 10 (-M 10). These parameters were chosen empirically based on the purpose of the analysis and corresponded to the consensus values giving consistent results for each taxonomic group. Matrix counts were normalized within each sample for comparative analysis and imported into R for statistical analysis and graphics generation. For oligotyping, we performed quality filtering of raw sequencing data generated by 454 sequencing technology. **Supplementary Table 2** summarizes all custom parameters utilized and the number of oligotypes generated for each taxa analyzed. Bacterial DNA sequences are available at DDBJ/EMBL/GenBank under the accession KAFK00000000.

### Statistical Analysis

Probability distribution of data was determined using the function descdist from the fitdistrplus package (Delignette-Muller and Dutang, 2015) and uniformity of variance was tested with the Levene test within the car package in R (Fox and Weisberg, 2011). The human groups for comparison included three healthy subjects, six IPF, and three lung cancer patients, and the murine groups included 7 WT and 12 TGF-β1 TG mice. Differences between experimental groups were evaluated using the Kruskal–Wallis test for continuous variables. We used the Wilcoxon rank sum test to evaluate differences in oligotype profiles in the R (package stats) between the study groups by sample source. Permutational multivariate analysis of variance (PERMANOVA) was performed to valuate variations in the dataset (Anderson, 2001). A *p*-value of <0.05 was considered as statistically significance.

## Results

### Microbial Communities at the Phylum and Genus Level

#### Luminal (BALF) and Saliva Samples

**FIGURE 1:**
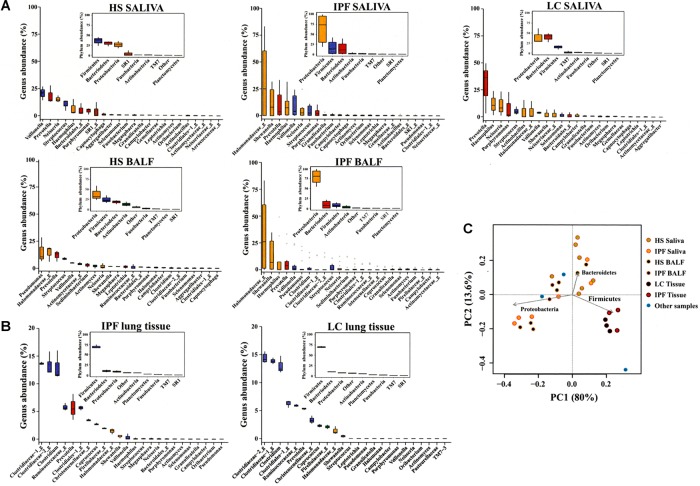
Community profiles of saliva, BALF, and lung tissue in controls and patients. Saliva, bronchoalveolar lavage fluid (BALF) **(A)**, and lung tissues **(B)** were sampled from IPF and LC patients, and saliva and BALF **(A)** from HS to isolate genomic DNA, amplify the V123 region of the bacterial 16S ribosomal RNA gene through high-throughput sequencing and taxonomic profiling. The abundance variation box plots as determined by read abundance for the 38 most abundant genera in IPF and LC patients are displayed and colored by taxonomic affiliation at the phylum level. Corresponding insets exhibit phylum abundance box plots. Principal component analysis (PcoA) was also calculated **(C)**. Saliva (*n* = 6), BALF (*n* = 3), and lung tissue (*n* = 3) samples were available from IPF patients; saliva (*n* = 3) and lung tissue (*n* = 3) samples were available from LC patients. “Other samples” described in **(C)** are BALF samples from patients with collagen vascular disease-associated interstitial lung disease (*n* = 2) and lung tissue sample from a patient with pneumothorax (*n* = 1). BALF, bronchoalveolar lavage fluid; IPF, idiopathic pulmonary fibrosis; LC, lung cancer; HS, healthy subjects.

Analysis of bacterial communities by 16S rRNA gene amplicon sequencing revealed a trend toward enhanced proportion of *Proteobacteria* in saliva and BALF of IPF patients compared to healthy subjects (**Figure 1A** and **Supplementary Tables 3, 4**). *Proteobacteria* tended to be predominant in saliva from lung cancer patients compared to healthy subjects. Subsequent evaluation at the genus level showed that *Halomonadaceae* and *Shewanellaceae*, both members of the *Proteobacteria*, tended to be more dominant in saliva and BALF from IPF patients compared to healthy subjects. The main genera detected in saliva and BALF of IPF patients were an unknown genus belonging to the *Halomonadaceae* (hereinafter referred to as *Halomonadaceae_g*), and *Shewanella, Prevotella, Haemophilus, Veillonella, Neisseria, Pseudomonas, Clostridium*, and *Streptococcus* (**Figure 1A** and **Supplementary Tables 3, 4**).

#### Tissue Samples

Pulmonary tissue samples from IPF and lung cancer patients displayed similar bacterial taxonomic profiles. Lung tissue samples were characterized by a large proportion of *Firmicutes* and a high percentage of unclassified bacteria (**Figure 1B** and **Supplementary Table 5**). There was a small proportion of *Bacteroidetes, Proteobacteria, Actinobacteria*, and other minority phyla. Main families within the *Firmicutes* included *Clostridiaceae, Lachnospiraceae, Ruminococcaceae*, two new families within the *Clostridiales*, and *Christensenellaceae*. A more detailed analysis showed two unknown *Clostridiaceae* genera (*Clostridiaceae-1_g, Clostridiaceae-2_g*) and *Clostridium* as the predominant genera detectable in lung tissue from IPF (*p* > 0.05) and lung cancer patients (**Figure 1B** and **Supplementary Table 5**). None of the differences among taxonomic groups between lung cancer and IPF patients were significant.

An overall evaluation of the phylogenetic diversity among human groups and between TGF-β1 and WT murine groups disclosed no significant difference (**Supplementary Tables 6, 7**). However, the microbial population of lung tissue samples showed a significant phylogenetic divergence compared to that of BALF and saliva samples (**Supplementary Table 8**).

### Principal Component Analysis

The principal component analysis (PCoA) revealed that variation among samples was mostly due to abundance of the main detected bacterial phyla, *Proteobacteria, Firmicutes*, and *Bacteroidetes* without clear associations to healthy and diseased states, but with a tendency toward an enrichment of *Proteobacteria* in saliva and BALF from IPF patients, and of *Firmicutes* in lung tissues from lung cancer and IPF patients (**Figure 1C**). In addition, the PCoA revealed clustering of tissue samples from both lung cancer and IPF subjects, indicating that lung tissue communities from either condition display similar bacterial taxonomic profiles (**Figure 1C**).

### Bacterial Oligotypes

#### Luminal and Saliva Samples

To further resolve the bacterial diversity below the genus level, microbial oligotypes of the most dominant genera were analyzed by sample source. Oligotypes of *Streptococcus, Haemophilus, Neisseria, Veillonella, Prevotella*, and *Pseudomonas* were identified mainly in airway luminal samples (BALF, saliva) (**Supplementary Figures 2A–C** and **Supplementary Table 2**).

#### Tissue Samples

**FIGURE 2:**
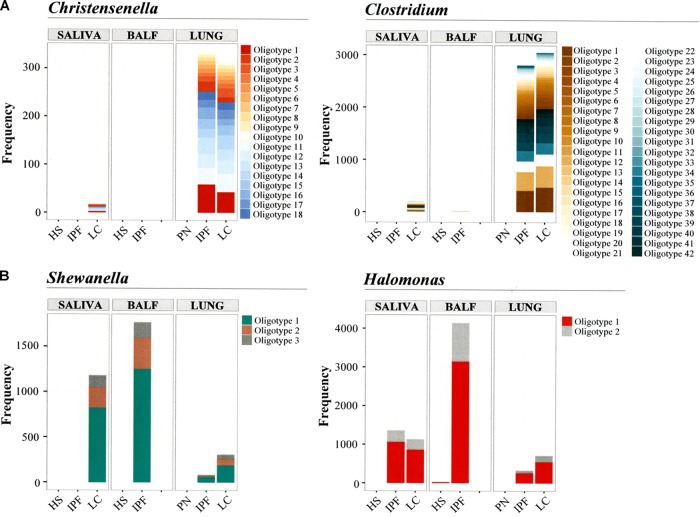
Oligotyping of microbial communities. Individual nucleotide positions were evaluated by Shannon entropy to identify the most frequent microbial community oligotypes across samples. Oligotypes of *Christensenella* and *Clostridium* **(A)**, and oligotypes of *Shewanella* and *Halomonas* **(B)** were detected. Saliva (*n* = 6), BALF (*n* = 3), and lung tissue (*n* = 3) samples were available from patients with IPF; saliva (*n* = 3) and lung tissue (*n* = 3) samples were available from LC patients. Lung tissue sample was available from one patient with PN. BALF, bronchoalveolar lavage fluid; HS, healthy subjects; PN, pneumothorax patient. Frequency in the *y*-axis represents the normalized oligotype counts.

*Clostridium* oligotypes were dominant in lung tissues from IPF and lung cancer patients (**Figures 2A,B**). Oligotypes of *Shewanella* and an undescribed bacteria of *Halomonadaceae* genus (*Halomonadaceae_g*) were detected in tissue samples from IPF and lung cancer patients, as well as in the luminal samples (**Figures 2A,B**). We also detected several oligotypes of unknown *Christensenellaceae genera (Christensenellaceae_g*). The most prevalent *Christensenellaceae_g* oligotype (i.e., oligotype 1, **Figure 2A**) in IPF and LC patients displayed the highest similarity with an uncultured member of the *Clostridiales* isolated from the rumen (**Supplementary Table 2**). The main oligotypes within *Clostridium* in lung tissues from IPF and lung cancer patients (oligotypes 1, 12, and 41; **Figure 2A** and **Supplementary Table 2**) shared similarities ranging from 99 to 100% with *Clostridium butyricum* strain JDY6D1. *Shewanella* oligotypes in lung tissues from IPF and lung cancer patients were predominated by oligotypes 1 and 2, which exhibited close to 100% similarity with the *Shewanella haliotis* strain 0315 (**Figure 2B** and **Supplementary Table 2**). These oligotypes also dominated in saliva and BALF from lung cancer and IPF patients. For the yet uncharacterized genus *Halomonadaceae_g*, the most detected oligotype (oligotype 1) showed 100% similarity with an uncultured *Halomonas* strain isolated from the intestinal microbiota of *Rutilus rutilus*, the common roach (**Figure 2B** and **Supplementary Table 2**). This oligotype was also detectable in saliva from both IPF and lung cancer patients in addition to BALF from IPF patients.

An overall evaluation of statistical differences in the oligotype profiles for *Christensenellaceae, Clostridium, Shewanella*, and *Halomonadaceae* was performed between the study groups (healthy subjects/pneumothorax, IPF, lung cancer) by sample source (saliva, BALF, lung tissue). The analysis showed that the oligotype profiles of *Clostridium* (*p* < 0.001) in saliva could significantly differentiate groups as compared to other sample sources. There was tendency but not significant difference by sample source in the oligotype profiles of *Shewanella* or *Halomonadaceae*, which was likely due to the low number of subjects within each group (**Supplementary Table 9**).

### Bacterial Populations in Aging TGF-β1 Transgenic Mouse With Pulmonary Fibrosis

**FIGURE 3:**
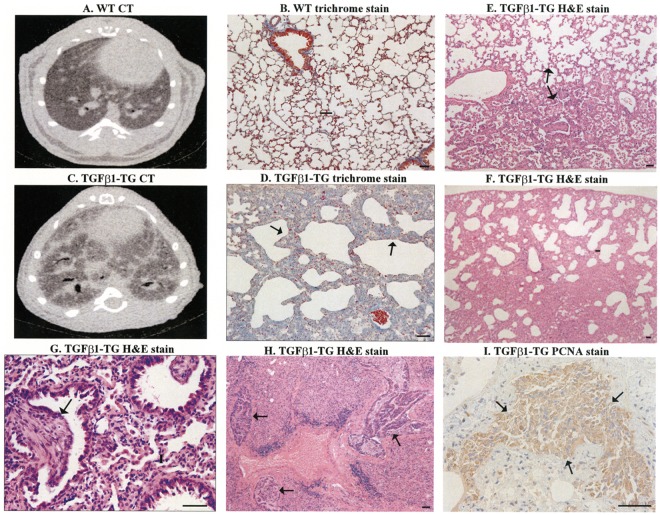
Pulmonary fibrosis in transgenic (TG) mice overexpressing human transforming growth factor (TGF)-β1. CT and trichrome stain of lung tissues from WT mice **(A,B)** and TGF-β1 mice (**C**,**D**, arrows). H&E stain showed areas of fibrotic changes alternate with areas with less affected parenchyma (**E**, arrows), honeycombing-like cysts with disruption of lung parenchyma **(F)**, and fibroblastic foci-like structures (**G**, arrow), and multiples areas with adenocarcinoma (**H**, arrows). There is increased staining of proliferating cell nuclear antigen (PCNA) in cancerous areas (**I**, arrows). Scale bars indicate 50 μm.

To demonstrate whether a fibrotic environment favors the growth of specific groups of lung microbiota, we evaluated the microbial communities in lung tissue from mice overexpressing the human profibrotic cytokine TGF-β1 specifically in the lungs (D’Alessandro-Gabazza et al., 2012). Similar to the disease in humans, aged (>30 weeks) TGF-β1 transgenic mice spontaneously develop pulmonary fibrosis with a progressive and fatal course (**Figures 3A–D**). The lung histopathologic findings are characterized by lung scarring, and honeycomb cysts alternating with areas of less affected parenchyma (**Figures 3D–F**). The lungs of transgenic mice also exhibit fibroblast foci-like areas and, at late stages, lung cancer complication (**Figures 3G–I**).

**FIGURE 4:**
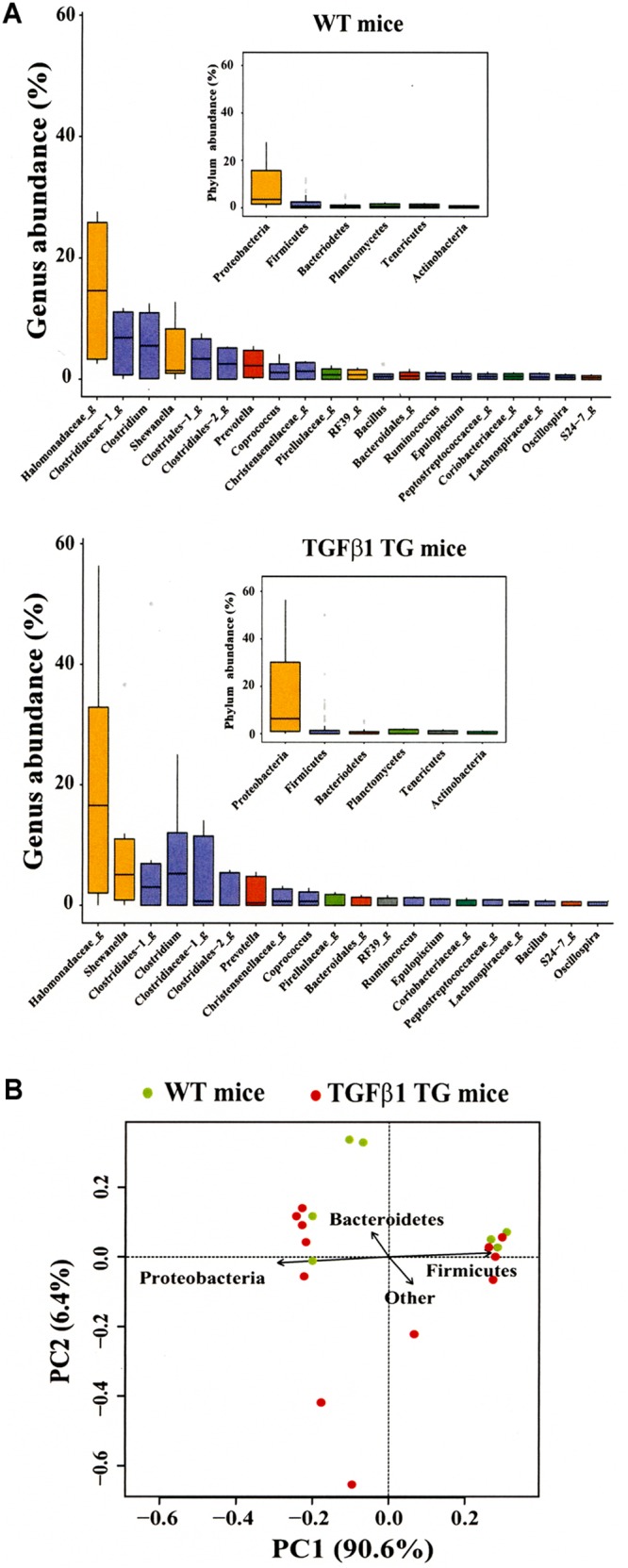
Community profiles of lung tissue in wild-type (WT) and TGF-β1 transgenic (TG) mice. Analysis of microbial composition of lung tissues from both WT and TGF-β1 TG mice at the phylum and genus level **(A)**. PCoA was also performed **(B)**.

Evaluation of bacterial composition based on 16S rRNA gene amplicon sequencing revealed relative abundance of *Proteobacteria* in both WT littermates and transgenic mice; however, at the genus-level, a trend toward an increase in members of *Halomonadaceae* and *Shewanellaceae* was observed in TGF-β1 transgenic mice compared to their WT counterparts (**Figure 4A**). The PCoA explained a 97% of the variation among samples, which was mostly related to abundance of *Proteobacteria* and *Firmicutes*, without clear associations between WT and TGF-β1 transgenic mice (**Figure 4B**).

### Alpha and Beta Diversities

The distribution pattern of the alpha diversity was displayed using the Faith’s phylogenetic diversity metric (Faith, 1992). There were no significant differences among the different human and mouse groups (**Supplementary Figures 3A,B**). Beta diversity of the human and mouse groups was evaluated above by PCoA of weighted unifrac distances and by PERMANOVA.

## Discussion

### Lung Sampling, Risk of Contamination, and Microbial Communities

Recent studies have demonstrated that the normal lung is not sterile and the existence of a healthy lung microbiome (Moffatt and Cookson, 2017). However, the risk of microbial contamination or false positive finding is still a major concern during lung molecular microbiome studies. Contamination can occur during specimen sampling, DNA extraction, amplicon preparation, and sequencing (Tanner et al., 1998; Salter et al., 2014). Bronchoscopy is generally used for sampling. During the procedure, a sterilized bronchoscope is advanced through the oral or nasal cavity, pharynx, and vocal cords before wedging into a middle or lingular lobe airway, thus during the procedure there is a risk of carrying contaminants from the oropharyngeal region to the lower respiratory tract (Han et al., 2014). Nonetheless, well-conducted investigations of bacterial topography in healthy human airways have demonstrated that contamination during oropharyngeal passage of the bronchoscope is actually minimum, thus providing validation for the use of bronchoscopy in lung microbiome studies (Charlson et al., 2011; Dickson et al., 2017). Based on the reported bacterial topography data we can assume that the lung luminal and tissue-resident microbes described here have migrated primarily by microaspiration and additionally by mucosal dispersion from the upper respiratory track (Dickson et al., 2017). The similar frequency of the BALF microbial communities at phylum level in healthy individuals and IPF patients between our findings and previous reports suggests that contamination was probably minimum in our present study (Molyneaux et al., 2017; Takahashi et al., 2018). The risk of contamination of lung tissues sampled from IPF and lung cancer patients, and of the lungs excised from WT and TGF-β1 TG mice was also minimum, at least during sampling, because sampling was performed under sterile surgical conditions. Interestingly, there was a predominance of *Firmicutes* in the lung tissue from both IPF and lung cancer patients, whereas *Proteobacteria* and *Firmicutes* were abundance in the lung tissue from human TGF-β1 TG mice. We have no explanation for the resemblance of the lung tissue microbial population between IPF and lung cancer patients. However, it is worth noting here that common genetic and epigenetic aberrations have been also reported in both IPF and lung cancer patients (Vancheri et al., 2010; Vancheri, 2013; Vancheri and du Bois, 2013; Stella et al., 2014; Vancheri, 2015).

### Oligotyping Versus Canonical Methods

In the present study, to test the hypothesis that a high resolution technique would identify so far undescribed microbial populations in the lungs, we used oligotyping, a computational method with high resolution, to evaluate the lung microbial community (Eren et al., 2013). Assignment of a microbial community analyzed by the 16S rRNA gene to a specific group is generally performed by comparing the DNA sequence to a reference database or by identifying OTUs from clustering sequences using an arbitrary similarity threshold (Wang et al., 2007; Huse et al., 2008, 2010; Liu et al., 2008; Schloss et al., 2009). However, each of these canonical approaches has important limitations. Reference-based classifications may fail to provide the real microbial diversity in a specific environment because of the lack of representative isolates in the database. On the other hand, the clustering approach may fail to identify microbial populations that differ from each other by small number of nucleotides, because researchers commonly use low similarity threshold to avoid random sequencing errors. Due to disadvantages of these canonical methods, we used here high-throughput sequencing followed by classification into groups that have identical sequences known as oligotypes. To determine oligotypes, the 16S rRNA gene sequences are aligned and the Shannon entropy is calculated to identify highly variable nucleotide positions. This high-resolution approach may allow the distinction between sequences differing by as little as a single nucleotide (Eren et al., 2014). In the present study, we performed oligotyping to clarify whether there are differential distribution patterns of oligotypes within our cohort groups. The results of this analysis disclosed the presence of several oligotypes of *Streptococcus, Haemophilus, Neisseria, Veillonella, Pseudomonas*, and *Prevotella* in BALF and saliva from both IPF and healthy subjects. More importantly, for the first time, various oligotypes of *Christensenella* and *Clostridium* were detected in lung tissue and a few oligotypes of *Shewanella* and *Halomonas* in both airway fluid and lung tissue from IPF and lung cancer patients, further suggesting the involvement of these microbial communities in fibrotic diseases.

### Proposal of a Mechanistic Hypothesis of IPF Based on Current Findings

Genome-wide association studies have shown that aberrations of several genes including surfactant protein, telomerases, and mucin are associated with high risk of developing IPF (Fingerlin et al., 2013). On the other hand, growing evidence indicates that host genetic variations can influence the microbiome composition (Goodrich et al., 2014; Blekhman et al., 2015; Luca et al., 2018). Therefore, it is conceivable that genetic alterations affect the lung microbial composition in patients with IPF. Here, we identified members of the *Christensenellaceae* family in lung fibrotic tissues. Previous study has shown that *Christensenella* is the most heritable microbe in the gut (Goodrich et al., 2014); that is, its presence or absence depends on the host genetic variant. In addition, the presence of *Christensenella* appears to predict the coexistence of other bacteria because it is apparently able to drive changes in the microbial community (Goodrich et al., 2014). The identification of *Christensenellaceae* family members here suggests their potential role in remodeling of lung microbial diversity in IPF. On the other hand, there is a consensus that IPF is triggered by repetitive lung epithelial injury causing enhanced activation and/or accelerated apoptosis of the alveolar lining epithelium and by increased lung recruitment of myofibroblasts leading to increased collagen production and deposition (King et al., 2011; Bagnato and Harari, 2015). Here we showed that *Halomonas* and *Shewanella* coexist in the IPF lung. Based on the halophilic and pro-apoptotic properties of *Halomonas* (Martinez-Canovas et al., 2004; Ruiz-Ruiz et al., 2011; Sagar et al., 2013) and sodium permeation-changing ability of *Shewanella* (Wang et al., 2008), we hypothesize that the presence of *Shewanella* increases extracellular levels of salt by blocking intracellular passage of sodium and thereby creating a propitious microenvironment for the growth of *Halomonas*. These halophilic bacteria secrete potent pro-apoptotic factors that may activate and subsequently induce increased apoptosis of alveolar epithelial cells (Ruiz-Ruiz et al., 2011; Sagar et al., 2013). Activated epithelial cells express several pro-fibrotic growth factors including TGF-β1, which may further stimulate the growth of *Halomonas* by promoting extracellular salt formation via inhibition of cell membrane expression of sodium and chloride channels (**Supplementary Figure 4**; Frank et al., 2003; Peters et al., 2014; Lutful Kabir et al., 2018).

The eventual detrimental role of a salty microenvironment in the pathophysiological scenario of IPF is supported by the following observations: (1) acute exacerbation of the disease in IPF patients is common following diagnostic bronchoalveolar lavage procedures in which a high volume of saline is used (Sakamoto et al., 2012), (2) increased circulating sodium chloride increases TGF-β1 expression (Hovater and Sanders, 2012), and (3) salt decreases the protective activity of mucin (Travis et al., 1999). The fact that mucin in turn can block the adverse effects of salt (Travis et al., 1999) may explain why IPF patients with a common risk polymorphism in *MUC5B*, associated with increased mucin production, have significantly improved survival (Peljto et al., 2013).

### Study Limitations

One limitation is the small size of the IPF and lung cancer population. It is generally difficult to obtain lung tissue samples from IPF patients because diagnostic bronchoscopy procedures are frequently associated with exacerbation or with fatal consequences of the fibrotic disease (Sakamoto et al., 2012).

## Conclusion

The discovery in the present study of a potentially pathogenic microbial community in direct association with lung tissue from IPF and TGF-β1 transgenic mice implicates this bacterial community in the pathogenesis of the fibrotic disease. The similarity between the microbial composition of the lung tissue from TGF-β1 TG mice with lung fibrosis and IPF suggests the potential usefulness of this mouse model for evaluating the role of the lung microbiome in the pathogenesis of IPF and for testing the therapeutic efficacy of antibiotics. However, further studies that examine tissues from a larger sample size and patients of different genetic backgrounds will help probe the observations made in this study. In addition, transplantation studies using TGF-β1 TG and germ-free mice would help confirm the impact of the lung microbiome in the pathogenesis of pulmonary fibrosis.

## Data Availability

Bacterial DNA sequences are available at DDBJ/EMBL/GenBank under the Accession No. KAFK00000000.

## Author Contributions

IC, ECG, and CND-G conceived the project, designed the study, wrote the manuscript, and supervised all phases of the investigation. IC, CM-G, SW, and RM performed the DNA sequencing and gene profiling. OH, FW, OT, TK, HF, and KF provided samples, interpreted clinical data, and supervised data analysis. MT, TY, and CND-G performed the sample collection and DNA extraction. KF and KN performed the microCT, genotyping, and additional microbial DNA extraction.

## Conflict of Interest Statement

ECG, CND-G, and IC have issued a patent on this new discovery. ECG and TK have a patent on the TGF-β1 TG mouse used in this study.The remaining authors declare that the research was conducted in the absence of any commercial or financial relationships that could be construed as a potential conflict of interest. The reviewer KB and handling Editor declared their shared affiliation.
