# Supplementary material for: Identification of Halophilic Microbes in Lung Fibrotic Tissue by Oligotyping
Source: Front Microbiol. 2018 Aug 30;9:1892. doi: 10.3389/fmicb.2018.01892 (PMC6127444; doi:10.3389/fmicb.2018.01892)
Supplement: Supplementary file 1 [file Data_Sheet_1.PDF]

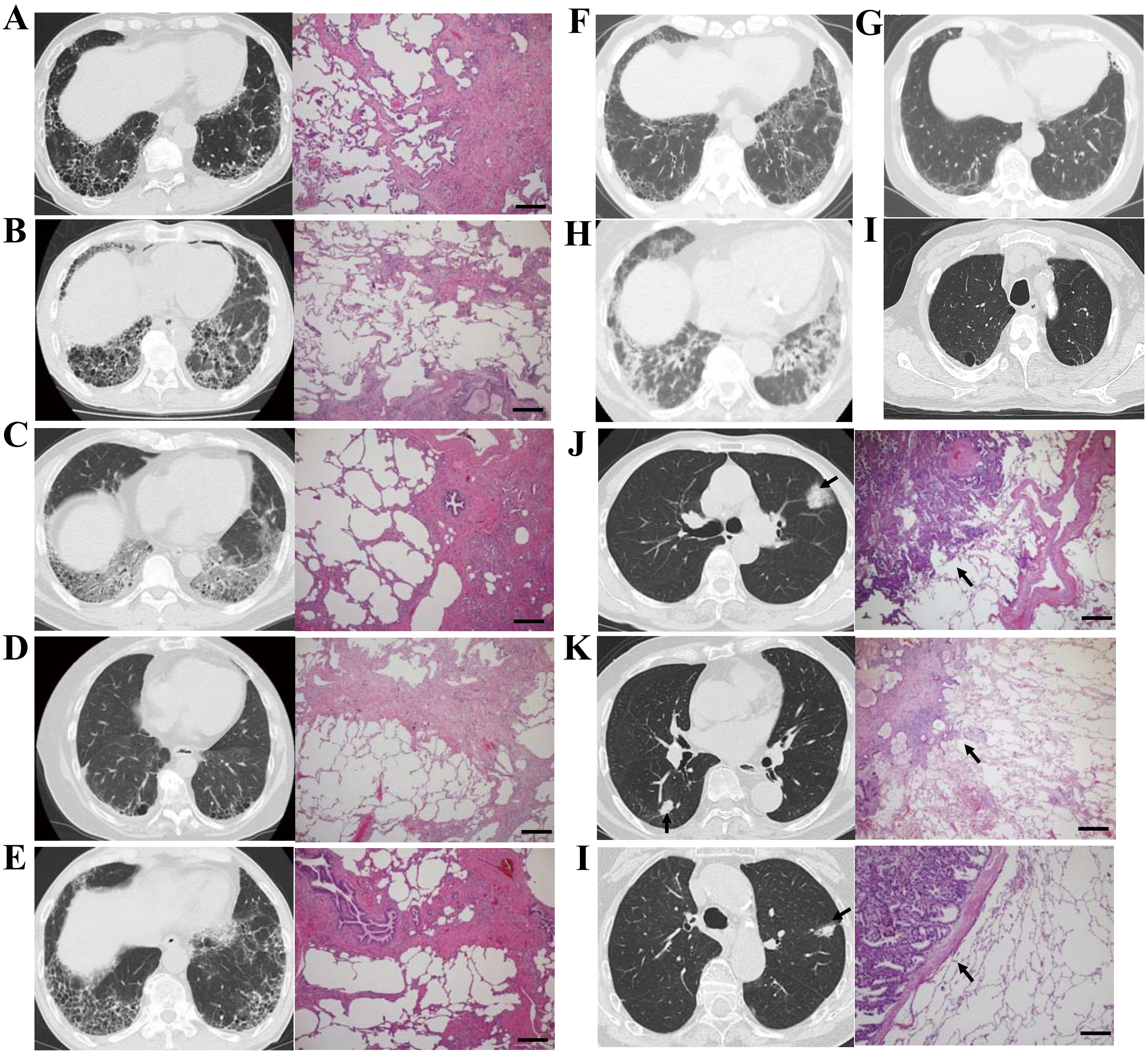

Supplementary Figure 1. High resolution computed tomography (HRCT) and histopathological findings in all subjects. HRCT and lung histopathological findings in idiopathic pulmonary fibrosis (IPF) cases 1 (**A**), 2 (**B**), 3 (**C**), 4 (**D**) and 5 (**E**). HRCT findings in IPF case 6 (**F**), collagen vascular disease-associated interstitial lung disease cases 1 (**G**) and 2 (**H**), and in the subject with pneumothorax (**I**). HRTC and lung histopathological findings in patients with lung adenocarcinoma cases 1 (**J**, arrow), 2 (**K**, arrow) and 3 (**L**, arrow)). Scale bars indicate 500 μm.
