# Supplementary material for: Identification of Halophilic Microbes in Lung Fibrotic Tissue by Oligotyping
Source: Front Microbiol. 2018 Aug 30;9:1892. doi: 10.3389/fmicb.2018.01892 (PMC6127444; doi:10.3389/fmicb.2018.01892)
Supplement: Supplementary file 2 [file Data_Sheet_2.PDF]

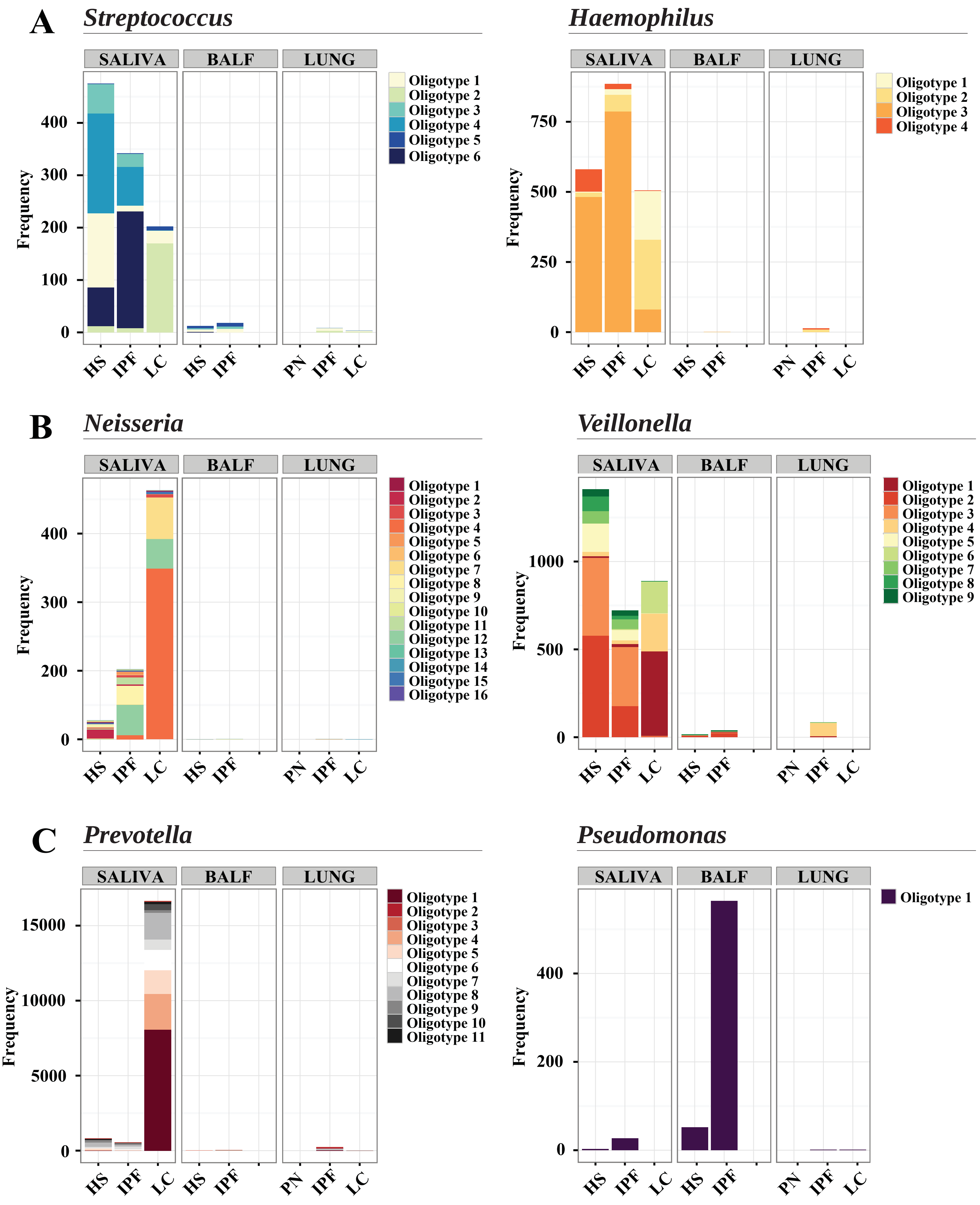

Supplementary Figure 2. Oligotypes of microbial communities preferentially detected in BALF and/or saliva samples. Oligotyping was performed based on the Shannon entropy to identify the most frequent microbial community oligotypes across the samples. *Streptococcus*, *Haemophilus*, *Neisseria*, *Veillonella* were abundant in saliva (A, B) of idiopathic pulmonary fibrosis (IPF), lung cancer (LC) and healthy subjects (HS). *Prevotella* were preferentially found in saliva from LC patients (C) and *Pseudomonas* (C) in bronchoalveolar lavage fluid (BALF) from IPF and HS. Frequency in the y-axis represents the normalized oligotype counts.
