# Supplementary material for: Identification of Halophilic Microbes in Lung Fibrotic Tissue by Oligotyping
Source: Front Microbiol. 2018 Aug 30;9:1892. doi: 10.3389/fmicb.2018.01892 (PMC6127444; doi:10.3389/fmicb.2018.01892)
Supplement: Supplementary file 3 [file Data_Sheet_3.PDF]

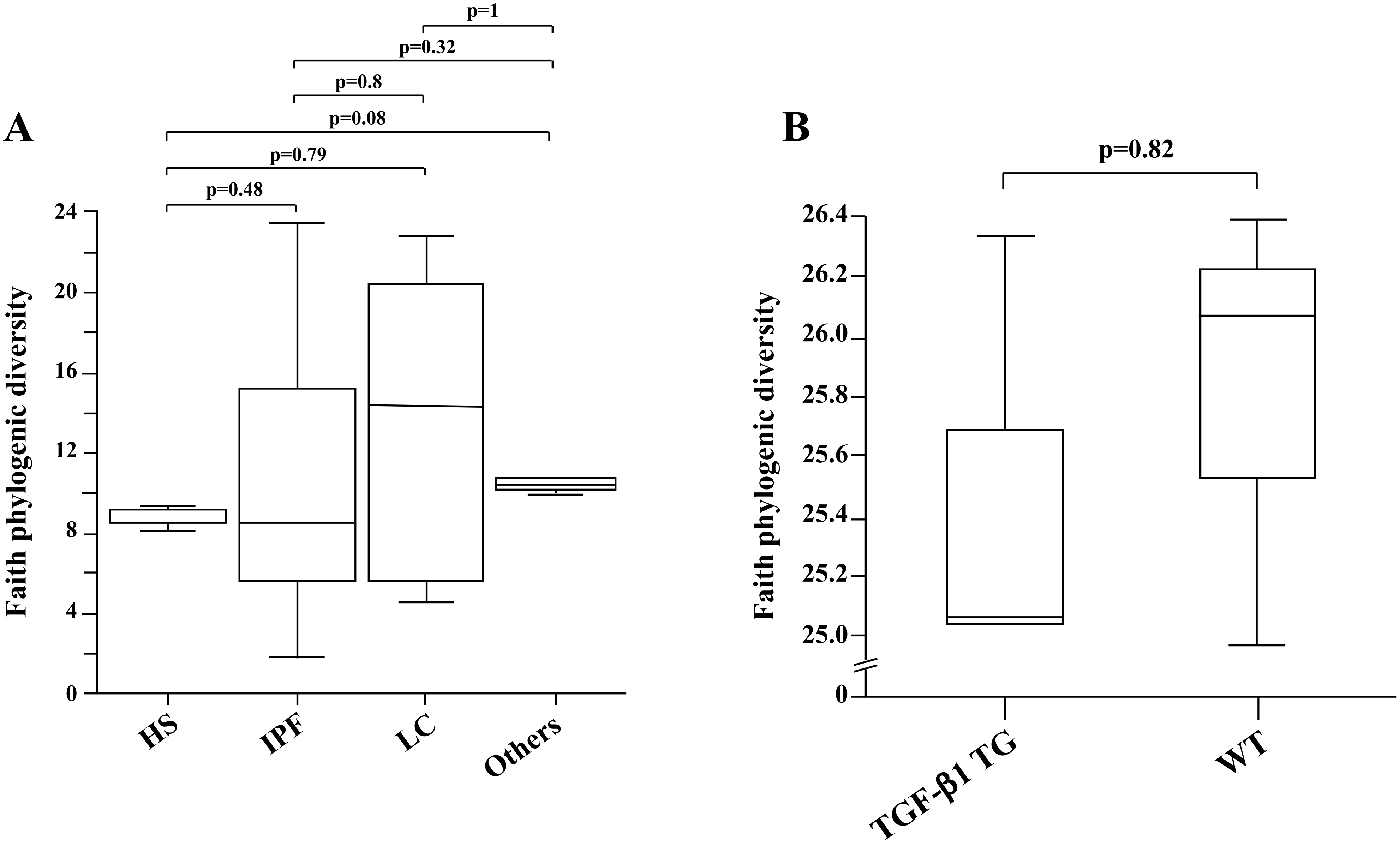

Supplementary Figure 3. The alpha diversity among human and murine groups. Boxplots of the alpha diversity in human (A) and mouse (B) groups displayed using the Faith's phylogenetic diversity metric. The p values from the Kruskal-Wallis analysis are shown. HS, human subjects; IPF, idiopathic pulmonary fibrosis; LC, lung cancer; others are samples from patients with collagen vascular disease-associated interstitial lung disease; TGF-β1 TG, transforming growth factor-β1 transgenic mice.
