# Supplementary material for: Identification of Halophilic Microbes in Lung Fibrotic Tissue by Oligotyping
Source: Front Microbiol. 2018 Aug 30;9:1892. doi: 10.3389/fmicb.2018.01892 (PMC6127444; doi:10.3389/fmicb.2018.01892)
Supplement: Supplementary file 4 [file Data_Sheet_4.PDF]

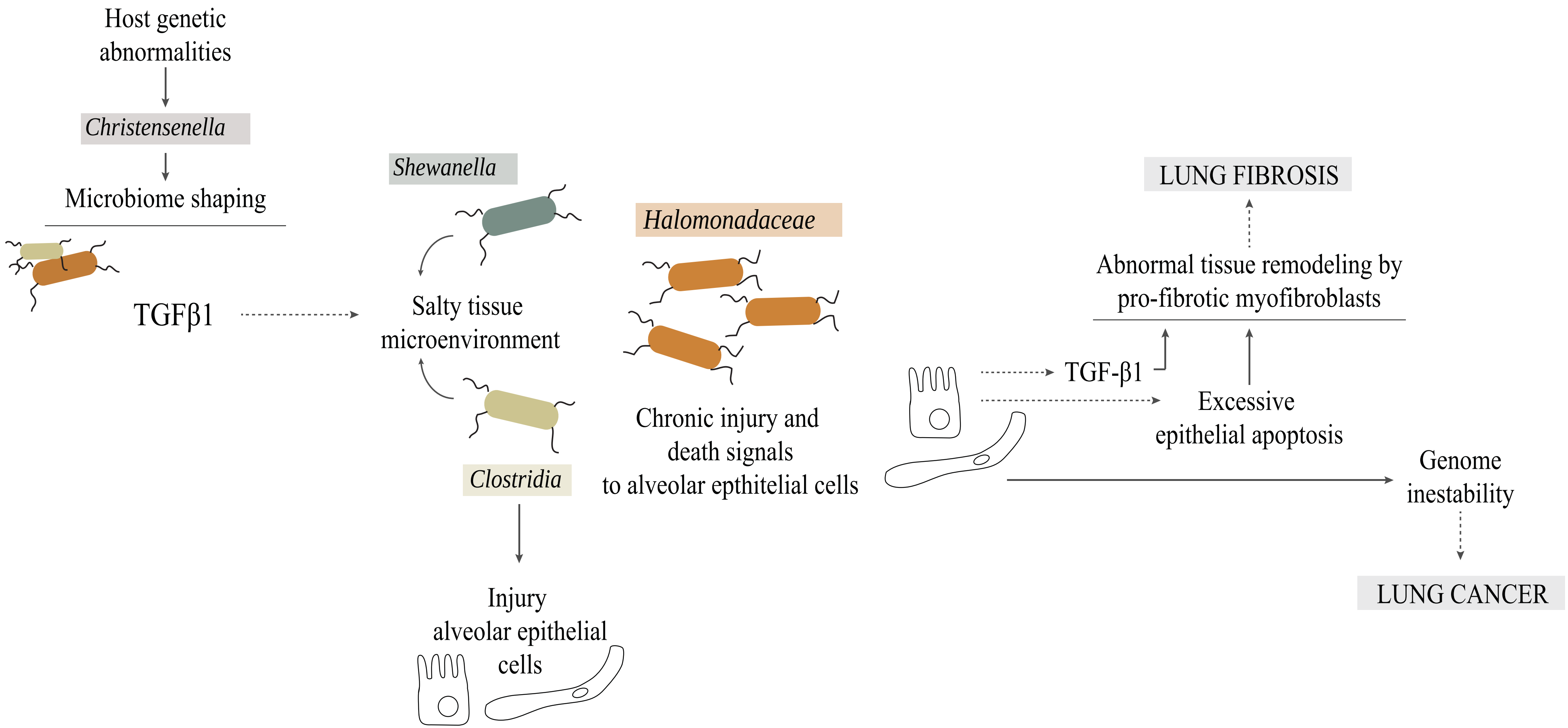

Supplementary Figure 4. Hypothesis on the role of the enriched microbiome in the pathogenesis of IPF. Host genetic abnormalities reshape the lung microbial population through members of the heritable taxon *Christensenella* family which together with a salty microenvironment favors the growth of *Halomonas*, *Shewanella* and *Clostridia*. Direct contact or secreted products from *Shewanella* and *Clostridia* can induce activation, while *Halomonas* can induce excessive apoptosis of alveolar lining epithelial cells. Transforming growth factor (TGF)-β1 that is secreted during epithelial activation can stimulate further growth by increasing salt in the microenviroment via inhibition of sodium channels. The increase in TGF-β1 levels in the lung tissue can enhance the lung population of collagen-producing myofibroblasts by promoting epithelial-mesenchymal transition, proliferation and chemotaxis of myofibroblasts. Genome instability may induce lung carcinogenesis.
