# Supplementary material for: Identification of Halophilic Microbes in Lung Fibrotic Tissue by Oligotyping
Source: Front Microbiol. 2018 Aug 30;9:1892. doi: 10.3389/fmicb.2018.01892 (PMC6127444; doi:10.3389/fmicb.2018.01892)
Supplement: Supplementary file 5 [file Table_1.DOCX]

**Supplemental Table 1. Characteristics of the subjects.**

Smoking

status

Diagnostic

method/therapy

Final

diagnosis

Anti-fibrotic

therapy

Subjects

Samples available

Age

M/F

Histology (VATS)

Histology (VATS)

Histology (VATS)

Histology (VATS)

Histology (VATS)

HRCT

Histology (Surgery)

Histology (Surgery)

Histology (Surgery)

HRCT

HRCT

Histology (VATS)

HRCT

HRCT

HRCT

Never smoked

Never smoked

Never smoked

Current smoker

Current smoker

Current smoker

Never smoked

Never smoked

Never smoked

Never smoked

Never smoked

Current smoker

Never smoked

Never smoked

Never smoked

IPF (UIP)

IPF (UIP)

IPF (UIP)

IPF

IPF

IPF

Adenocarcinoma

Adenocarcinoma

Adenocarcinoma

CDV-ILD

CVD-ILD

Pneumothorax,

bullae, normal tissue

Normal

Normal

Normal

Left lower lobe lung tissue, saliva

Left lower lobe lung tissue, saliva

Left lower lobe lung tissue, saliva

BALF (right middle lobe), saliva

BALF (right middle lobe), saliva

BALF (right middle lobe), saliva

Left upper lobe lung tissue, saliva

Left upper lobe lung tissue, saliva

Left upper lobe lung tissue, saliva

BALF (right middle lobe), saliva

BALF (right middle lobe), saliva

Right upper segmentectomy, saliva

BALF (right middle lobe), saliva

BALF (right middle lobe), saliva

BALF (right middle lobe), saliva

(-)

(-)

(-)

(-)

(+)

(+)

(-)

(-)

(-)

(-)

(-)

(-)

(-)

74

59

66

72

59

72

85

82

79

72

73

71

37

31

35

Adenocarcinoma

Adenocarcinoma

Adenocarcinoma

Not applicable

Not applicable

Not applicable

M

M

M

M

F

M

F

M

F

F

F

M

M

M

M

M

Adenocarcinoma

Adenocarcinoma

Adenocarcinoma

Not applicable

Not applicable

Not applicable

IPF patients

Case 1

Case 2

Case 3

Case 4

Case 5

Case 6

Lung cancer patients

Case 1

Case 2

Case 3

CVD-ILD

Case 1

Case 2

Pneumothorax

Case 1

Healthy subjects

Subject 1

Subject 2

Subject 3

IPF, idiopathic pulmonary fibrosis; UIP, usual interstitial pneumonitis; HRCT, high resolution computd tomography; VATS, video-assisted thoracic surgery; BALF, bronchoalveolar lavage fluid; CVD-ILD, collagen vascular disease-associated interstitial lung disease.
