# Supplementary material for: Identification of Halophilic Microbes in Lung Fibrotic Tissue by Oligotyping
Source: Front Microbiol. 2018 Aug 30;9:1892. doi: 10.3389/fmicb.2018.01892 (PMC6127444; doi:10.3389/fmicb.2018.01892)
Supplement: Supplementary file 6 [file Table_2.DOCX]

**Supplementary Table 2. Characteristics of microbial oligotypes in lung samples**

**BLAST hit**

**Oligotype**

**Accession**

**Number**

**Identity**

**(%)**

**RDP hit**

**S ab RDP**

**score**

**Source**

*Streptococcus*

1

2

3

4

5

6

*Haemophilus*

1

2

3

4

*Neisseria*

1

2

3

4

5

6

7

8

9

10

11

12

13

14

15

16

Porcine clinical specimen

Human Skin microbiome

Human digestive tract

Human skin microbiome

Dental plaque

Human microbiome

Healthy human colon

Offshore drilling core-sample

Human skin microbiome of patients with primary immunodeficiencies

mucosa-associated microbial communities of human colon

microbiota of red palm weevil

human skin microbiome in patients with primary immunodeficiencies

human skin microbiome in patients with primary immunodeficiencies

microbiota of red palm weevil

Commensal Neisseria

human skin microbiome in patients with primary immunodeficiencies

Gut microbiota of migratory passerines in the Gulf of Mexico

Korean oral cavity

human skin microbiome in patients with primary immunodeficiencies

The human mouth

The human skin microbiome

human skin microbiome in patients with primary immunodeficiencies

Model pipeline flow cell

Healthy oral cavity

Human mouth

Human mouth

KR819488

JF089367

CP013216

HM289315

KM225751

JX520259

HQ236904

AB806768

KF079074

KF088626

LN623629

KF085770

AB904058

LN623630

AB903974

KF108067

KT772438

KF733721

KF100557

JQ449394

JF189435

KF104026

JQ316615

FJ470633

JQ456579

JQ446645

*Streptococcus mitis* strain SS233

Uncultured bacterium clone ncd1282a06c1

*Streptococcus salivarius* strain HSISS4

Uncultured bacterium clone ncd753c01c1

*Streptococcus sp.* C12

*Streptococcus sp.* HXF0012

Uncultured bacterium clone 382B01

Uncultured bacterium

Uncultured bacterium clone nck223b01c1

Uncultured bacterium clone nck242e04c1

*Neisseria sp*. 260

Uncultured bacterium clone ncm46b05c1

*Neisseria perflava*

*Neisseria subflava*

*Neisseria flava*

Uncultured bacterium clone ncm66d08c1

Uncultured bacterium clone GCSTWT_16S_4114

*Neisseria flavescens* strain ChDC B580

Uncultured bacterium clone ncd1965c09c1

Uncultured bacterium clone 069104_089

Uncultured bacterium clone ncd2171e08c2

Uncultured bacterium clone nck276a06c1

Uncultured bacterium clone 2Ab07

Uncultured bacterium clone B_M_03_38

Uncultured bacterium clone 069102_380

Uncultured bacterium clone 069056_044

100

100

100

100

99

99

100

100

100

100

100

100

100

100

100

100

100

100

100

100

99

100

99

99

100

100

*Streptococcus mitis* (T)

uncultured *Streptococcus bacterium*

*Streptococcus salivarius* (T)

*Streptococcus pneumoniae*

*Streptococcus oralis*

uncultured

uncultured gamma *proteobacterium*

*Haemophilus parainfluenzae*

*Haemophilus haemolyticus*

uncultured *Haemophilus sp*.;

402F12(oral); AM420152

*Neisseria perflava*

uncultured *Neisseria bacterium*

*Neisseria subflava* (T)

uncultured *Neisseria bacterium*

uncultured *Neisseria bacterium*

"uncultured *Neisseria bacterium*"

*Neisseria mucosa*

uncultured *Neisseria bacterium*

Neisseria sp. oral clone AP085

uncultured *Neisseria sp.*

*Neisseria subflava* (T)

*Neisseria elongata;* I4; AJ239303

uncultured *Neisseria bacterium*

*Neisseria sp*. R-22841

uncultured *Neisseria bacterium*

uncultured *Neisseria bacterium*

1

0.97

1

0.968

0.968

0.968

1

1

1

0.975

0.968

0.97

1

1

0.968

1

0.947

0.968

0.949

0.747

0.967

0.952

**Supplementary Table 2. Characteristics of microbial oligotypes in lung samples (continued)**

*Veillonella*

1

2

3

4

5

6

7

8

9

*Prevotella*

1

2

3

4

5

6

7

8

9

10

11

12

*Pseudomonas*

1

**Source**

**S ab RDP**

**score**

**RDP hit**

**Identity**

**(%)**

99

99

100

100

99

100

99

99

99

100

100

100

100

100

100

100

99

100

100

100

100

100

**Accession**

**Number**

KF067956

KF093821

JQ449563

KT770143

KF092539

KF104620

JQ473290

KF080643

KF842683

HG818386

KF109721

JF204183

KF092223

HG818386

KF271038

KF099946

KU321258

JQ467555

KF092436

JX515819

JQ460190

KU323871

**Oligotype**

**BLAST hit**

human skin microbiome associated with primary immunodeficiencies

human skin microbiome associated with primary immunodeficiencies

Human mouth

gut microbiota migratory passerines

human skin microbiome associated with primary immunodeficiencies

human skin microbiome associated with primary immunodeficiencies

Human mouth

human skin microbiome associated with primary immunodeficiencies

fecal microbiota in a southern Indian rural population

bacterial communities related with meat and food spoilage

human skin microbiome in primary immunodeficiencies

skn microbiome related to atopic dermatitis

human skin microbiome in primary immunodeficiencies

bacterial communities related with meat and food spoilage

The West pacific

human skin microbiome in primary immunodeficiencies

bacterial populations on brewery filling hall surfaces

The human mouth

human skin microbiome in primary immunodeficiencies

Environmental, but where nnknown

The human mouth

leaf surface biodiversity of mulberry leaves shot-hole disease

0.966

0.979

0.968

0.968

1

0.97

0.968

0.968

0.968

1

1

0.983

1

0.968

0.985

1

0.995

1

1

1

0.941

0.97

Uncultured Veillonella bacterium

Veillonella sp. oral clone VeillC8

uncultured Selenomonas sp.

(family Veillonellaceae)

Veillonella dispar

uncultured Veillonella sp.

Uncultured Veillonella bacterium

Uncultured Veillonella bacterium

Uncultured Veillonella bacterium

Uncultured Veillonella bacterium

uncultured *Prevotella bacterium*

*Prevotella melaninogenica*

uncultured *Prevotella bacterium*

*Prevotella sp*. oral clone BE073

uncultured *Prevotella bacterium*

*Prevotella salivae* (T)

uncultured *Prevotella bacterium*

*Prevotella melaninogenica*

uncultured *Prevotella bacterium*

uncultured *Prevotella bacterium*

*Prevotella sp*. T05-04

*Prevotella sp*. oral clone GI032

*Pseudomonas sp*. M13

Uncultured bacterium clone nbw894e06c1

Uncultured bacterium clone nck182h03c1

Uncultured bacterium clone 069105_088

Uncultured bacterium clone GCSTWT_16S_1698

Uncultured bacterium clone nck164e05c1

Uncultured bacterium clone nck284h09c1

Uncultured bacterium clone 071059_495

Uncultured bacterium clone nck13f04c1

Uncultured bacterium clone PS_B193

Uncultured bacterium partial 16S rRNA gene, clone EBP1148

Uncultured bacterium clone nck335a01c1

Uncultured bacterium clone ncd2331d02c1

Uncultured bacterium clone nck160b03c1

Uncultured bacterium partial 16S rRNA gene, clone EBP1148

Uncultured bacterium clone N8_12_C_OH_35

Uncultured bacterium clone nck262d04c1

*Prevotella melaninogenica* strain VTT E-052771

Uncultured bacterium clone 070053_279

Uncultured bacterium clone nck163c07c1

Uncultured bacterium clone HE1S21F01DLFQP

Uncultured bacterium clone 070054_028

*Pseudomonas oryzihabitans* strain MB03

**Supplementary Table 2. Characteristics of microbial oligotypes in lung samples (continued)**

**RDP hit**

**Identity**

**(%)**

**Accession**

**Number**

**Source**

**S ab RDP**

**score**

**Oligotype**

**BLAST hit**

Uncultured bacterium clone J2_1_2746

Uncultured bacterium clone RL186_aan92b09

Uncultured bacterium clone J2_1_2746

Uncultured bacterium clone SP3_a07

Uncultured sludge bacterium clone ASB34

Uncultured sludge bacterium clone ASB34

Uncultured bacterium clone DXC-22

Uncultured bacterium clone J2_1_2746

Uncultured bacterium clone DF5988

Uncultured bacterium clone Wu-C98

Uncultured bacterium clone J2_1_2654

Uncultured bacterium clone BAZ_d03

Uncultured bacterium clone BAZ_d03

Uncultured bacterium clone GDIC2IK01DKJO0

Uncultured bacterium clone XXC-71

Uncultured bacterium 054H02_B_DI_P58

Uncultured bacterium clone

Pohang_WWTP_August.2006_6218

Uncultured bacterium clone PBg1-091

*Clostridium butyricum* strain JKY6D1

Uncultured bacterium clone AHPD_Bac4_A11

Uncultured bacterium clone p-2171-s959-3

Uncultured bacterium clone AHPD_Bac4_A11

Uncultured bacterium clone AHPD_Bac4_A11

Uncultured bacterium clone AHPD_Bac4_A11

JQ150640

DQ806422

JQ150640

EU469134

FJ947133

FJ947133

KJ957652

JQ150640

GU614165

KJ783174

JQ163651

EU467297

EU467297

JF579461

KJ957730

CR933204

HQ503168

AY791216

CP013352

KJ206844

AF371841

KJ206844

KJ206844

KJ206844

97

95

97

94

95

95

98

97

98

95

95

95

93

96

96

96

93

93

100

100

98

99

99

99

0.757

0.719

0.757

0.682

0.821

0.821

0.809

0.757

0.797

0.897

0.711

0.732

0.732

0.724

0.799

0.807

0.705

0.824

1

0.981

0.779

0.981

0.981

0.981

Anaerobic sludge digester

Human gut microbe associated with dabetes

Anaerobic digester

Speke’s gazelle feces

Activated sludge

Activated sludge

Cow manure

Activated sludge

Dairy cow fecal samples

from the petrochemical wastewater treatment plant activated sludge

anaerobic digesters

gut microbe

gut microbe

anaerobic digestion of carrot waste

Cow manure

anaerobic digester

activated sludge

From insect gut

Unknown

Lab scale bioreactor

The swine intestine

Lab scale bioreactor

Lab scale bioreactor

Lab scale anaerobic bioreactor

*Christensenella*

1

2

3

4

5

6

7

8

9

10

11

12

13

14

15

16

17

18

*Clostridium*

1

2

3

4

5

6

uncultured *Clostridiales rumen bacterium*

uncultured *Ruminococcaceae bacterium*

uncultured *Clostridiales rumen bacterium*

uncultured *Clostridiales bacterium*

uncultured *Clostridiales rumen bacterium*

uncultured *Clostridiales rumen bacterium*

uncultured *Clostridiales rumen bacterium*

uncultured *Clostridiales rumen bacterium*

uncultured *Clostridiales rumen bacterium*

uncultured *Clostridiales rumen bacterium*

uncultured *Clostridiales rumen bacterium*

uncultured *Clostridiales rumen bacterium*

uncultured *Clostridiales rumen bacterium*

uncultured *Clostridiales rumen bacterium*

uncultured *Clostridiales rumen bacterium*

uncultured *Clostridiales rumen bacterium*

uncultured *Clostridiales bacterium*

uncultured *Clostridiales bacterium*

*Clostridium butyricum*

*Clostridium butyricum*

*Clostridium baratii*

*Clostridium butyricum*

*Clostridium butyricum*

*Clostridium butyricum*

**Supplementary Table 2. Characteristics of microbial oligotypes in lung samples (continued)**

**S ab RDP**

**score**

**Source**

**RDP hit**

**Identity**

**(%)**

**Accession**

**Number**

**Oligotype**

**BLAST hit**

*Clostridium*

7

8

9

10

11

12

13

14

15

16

17

18

19

20

21

22

23

24

25

26

27

28

29

30

31

32

33

Gut of earth worm

Swine waste

Feces of a pig

Municipal solid waste

Anaerobic digester sludge

Pit mud of a Chinese liquor factory

Pit mud of a Chinese liquor factory

Pit mud of a Chinese liquor factory

Pit mud of a Chinese liquor factory

Dugong feces

Lab scale bioreactor

Lab scale bioreactor

Swine waste

Lab scale bioreactor

Swine intestine

Human skin microbiome

Feces of a dugong

Paddy soil enrichment

Pit mud of a Chinese liquor factory

Gut contents of the earth worm

Fecal contents of a matured pig

Swine intestine

Swine intestine

Fresh water sediments

Human skin microbiome (Disease)

Anaerobic sludge

Lab scale anaerobic bioreactor

0.917

0.94

0.899

0.882

0.947

0.984

0.979

0.986

0.963

0.947

0.954

0.963

0.956

0.934

0.952

0.963

0.917

0.911

0.984

0.897

0.899

0.991

0.954

0.931

0.945

0.928

0.956

*Clostridium butyricum*

uncultured *Clostridium anaerobic bacterium*

uncultured *Clostridium bacterium*

uncultured bacterium

uncultured *Clostridiaceae bacterium*

*Clostridium butyricum*

*Clostridium butyricum*

*Clostridium butyricum*

*Clostridium butyricum*

*Clostridium butyricum*

*Clostridium butyricum*

*Clostridium butyricum*

uncultured *Clostridium bacterium*

*Clostridium butyricum*

uncultured *Clostridiaceae bacterium*

uncultured *Clostridiaceae bacterium*

uncultured *Clostridiaceae bacterium*

uncultured *Clostridiaceae bacterium*

*Clostridium butyricum*

uncultured *Clostridium bacterium*

uncultured *Clostridium bacterium*

uncultured *Clostridium bacterium*

uncultured *Clostridium bacterium*

uncultured *Clostridium bacterium*

*Clostridium butyricum*

*Clostridium butyricum*

*Clostridium butyricum*

98

99

97

98

99

99

99

99

99

99

99

99

99

99

99

99

99

99

99

99

97

99

99

98

99

99

99

HG964590

KT834728

AB506292

GQ453625

KJ853880

CP013352

CP013352

CP013352

CP013352

AB218317

KJ206844

KJ206844

GQ136671

KJ206844

AF371841

KF071407

AB218319

JX473610

CP013352

HG964590

AB506292

AF371841

AF371841

KC441671

HM327668

KJ853880

KJ206844

Uncultured bacterium partial 16S rRNA gene, clone BE12L_BC9

Uncultured bacterium isolate DGGE gel band RA2-33

Uncultured bacterium gene for 16S rRNA, partial sequence,

clone: R-C-A09.

Uncultured bacterium clone 828_39_pH6.2

Uncultured bacterium clone 3N21hL107

*Clostridium butyricum* strain JKY6D1

*Clostridium butyricum* strain JKY6D1

*Clostridium butyricum* strain JKY6D1

*Clostridium butyricum* strain JKY6D1

Uncultured *Clostridiaceae bacterium* gene for 16S rRNA,

partial sequence, clone: dgB-113

Uncultured bacterium clone AHPD_Bac4_A11

Uncultured bacterium clone AHPD_Bac4_A11

Uncultured bacterium clone 4f09

Uncultured bacterium clone AHPD_Bac4_A11

Uncultured bacterium clone p-2171-s959-3

Uncultured bacterium clone ncd478g11c1

Uncultured *Clostridiaceae bacterium* dgB-84

Bacterium enrichment culture clone HY357

*Clostridium butyricum* strain JKY6D1

Uncultured bacterium BE12L_BC9

Uncultured bacterium R-C-A09

Uncultured bacterium clone p-2171-s959-3

Uncultured bacterium clone p-2171-s959-3

Bacterium enrichment culture clone ZZ_A6

Uncultured bacterium clone ncd480a02c1

Uncultured bacterium clone 3N21hL107

Uncultured bacterium clone AHPD_Bac4_A11

**Supplementary Table 2. Characteristics of microbial oligotypes in lung samples (continued)**

**Source**

**S ab RDP**

**score**

**RDP hit**

**Oligotype**

**Accession**

**Number**

**BLAST hit**

**Identity**

**(%)**

*Clostridium*

34

35

36

37

38

39

40

41

42

*Shewanella*

1

2

3

*Halomonadaceae*

1

2

Human gastrointestinal specimen

Pit mud of a Chinese liquor factory

Lab scale bioreactor

Swine waste

Gut contents of earth worm

Skin microbiome associated with disease

Gut contents of the earth worm

Pit mud of a Chinese liquor factory

Lab scale bioreactor

Marine seagrass

Marine sea grass

Marine sea grass

Intestinal microbial community of roach

Intestinal microbial community of roach

0.879

0.943

0.934

0.945

0.918

0.915

0.911

0.974

0.909

1

1

0.985

0.944

0.944

*Clostridium subterminale*

*Clostridium butyricum*

*Clostridium butyricum*

uncultured *Clostridium bacteriu*m

*Clostridium butyricum*

uncultured *Clostridium bacterium*

uncultured *Clostridium bacterium*

*Clostridium butyricum*

*Clostridium butyricum*

*Shewanella sp.* A6.mk

*Shewanella sp*. A6.mk

*Shewanella sp*. L-10

*Halomonas sp*. A-2

*Halomonas sp*. A-3

99

98

100

99

99

99

99

99

97

100

100

100

100

100

HQ800034

CP013352

KJ206842

GQ136671

HG964590

HM267218

HG964590

CP013352

KJ206844

KP236430

KP236430

KP236204

KP967468

KP967482

Uncultured organism clone ELU0145-T222-S-NIPCRAMgANa_000131

*Clostridium butyricum* strain JKY6D1

Uncultured bacterium clone AHPD_Bac4_A09

Uncultured bacterium clone 4f09

Uncultured bacterium BE12L_BC9

Uncultured bacterium clone ncd211e04c1

Uncultured bacterium BE12L_BC9

*Clostridium butyricum* strain JKY6D1

Uncultured bacterium clone AHPD_Bac4_A11

Shewanella haliotis strain 0315

Shewanella haliotis strain 0315

Shewanella chilikensis strain 0066

Uncultured Halomonas sp. clone Z2_KL_433-12

Uncultured Halomonas sp. clone S_KL_373-12
