# Supplementary material for: Identification of Halophilic Microbes in Lung Fibrotic Tissue by Oligotyping
Source: Front Microbiol. 2018 Aug 30;9:1892. doi: 10.3389/fmicb.2018.01892 (PMC6127444; doi:10.3389/fmicb.2018.01892)
Supplement: Supplementary file 7 [file Table_3.DOC]

**Supplementary Table 3. Genus frequency in saliva (%)**

**ance in BALF**

**Healthy subjects**

**(n=3)**

**Genera**

**IPF patients (n=3)**

**Other**

**(n=2)**

**LC patients (n=3)**

*g__Actinobacillus*

*g__Actinomyces*

*g__Actinomycetaceae*

*g__Aerococcaceae*

*g__Aggregatibacter*

*g__Bacteroidales*

*g__Campylobacter*

*g__Capnocytophaga*

*g__Christensenellaceae*

*g__Clostridiaceae-1*

*g__Clostridiaceae-2*

*g__Clostridiales-1*

*g__Clostridium*

*g__Coprococcus*

*g__Fusobacterium*

*g__Gemellaceae*

*g__Granulicatella*

*g__Haemophilus*

*g__Halomonadaceae*

*g__Halomonas*

*g__Leptotrichia*

*g__Megasphaera*

*g__Neisseria*

*g__Neisseriaceae*

*g__Oribacterium*

*g__Pasteurellaceae*

*g__Pirellulaceae*

*g__Porphyromonas*

*g__Prevotella*

*g__Pseudomonas*

*g__Ruminococcaceae*

*g__Sediminibacterium*

*g__Selenomonas*

*g__Shewanella*

*g__SR1*

*g__Streptococcus*

*g__TM7-3*

*g__Veillonella*

0.373 ± 0.6460

0.505 ± 0.1760

0.210 ± 0.2420

0.034 ± 0.0300

0.954 ± 0.5320

5.322 ± 5.4520

0.652 ± 0.1520

1.292 ± 1.2410

0.000 ± 0.0000

0.000 ± 0.0000

0.000 ± 0.0000

0.292 ± 0.2870

0.000 ± 0.0000

0.003 ± 0.0050

0.736 ± 0.2210

0.557 ± 0.0260

0.700 ± 0.1860

6.922 ± 7.3960

0.003 ± 0.0050

0.000 ± 0.0000

0.529 ± 0.1270

0.729 ± 0.8350

15.258 ±3.0890

0.140 ± 0.0380

0.411 ± 0.0250

0.022 ± 0.0220

0.000 ± 0.0000

4.469 ± 2.9520

18.420 ± 8.2580

0.013 ± 0.0130

0.000 ± 0.0000

0.009 ± 0.0150

0.880 ± 0.4800

0.000 ± 0.0000

4.264 ± 7.1540

10.582 ± 5.1360

0.000 ± 0.0000

20.687± 6.3530

0.243 ± 0.5920

0.390 ± 0.5260

0.059 ± 0.0810

0.001 ± 0.0030

0.021 ± 0.0520

0.241 ± 0.3290

0.635 ± 0.7380

0.467 ± 0.5850

0.000 ± 0.0000

0.000 ± 0.0000

0.000 ± 0.0000

0.108 ± 0.0980

0.020 ± 0.0480

0.000 ± 0.0000

0.861 ± 0.9110

0.279 ± 0.4000

1.039 ± 1.2920

10.055 ± 12.1530

32.830 ± 37.3610

0.000 ± 0.0000

0.309 ± 0.3770

0.300 ± 0.2770

6.329 ± 9.5690

0.074 ± 0.1190

0.348 ± 0.4870

0.016 ± 0.0390

0.000 ± 0.0000

3.231 ± 4.1530

10.818 ± 14.4160

0.156 ± 0.2690

0.000 ± 0.0000

0.066 ± 0.1050

0.344 ± 0.5170

12.423 ± 14.5300

0.227 ± 0.4400

4.485 ± 5.2230

0.000 ± 0.0000

9.220 ± 11.4100

5.207 ± 8.9380

0.660 ± 0.9750

0.090 ± 0.1010

0.010 ± 0.0170

0.025 ± 0.0370

1.495 ± 2.5320

1.402 ± 1.7800

0.321 ± 0.3550

0.000 ± 0.0000

0.005 ± 0.0080

0.000 ± 0.0000

0.262 ± 0.4540

0.001 ± 0.0020

0.013 ± 0.0220

0.822 ± 0.4090

0.347 ± 0.2210

0.924 ± 0.6680

12.007 ± 11.3130

5.123 ± 8.2890

0.000 ± 0.0000

0.264 ± 0.2340

0.543 ± 0.4430

10.926 ± 11.4900

2.311 ± 3.9680

0.544 ± 0.7210

0.000 ± 0.0000

0.000 ± 0.0000

8.237 ± 12.7720

29.558 ± 23.8250

0.000 ± 0.0000

0.002 ± 0.0030

0.000 ± 0.0000

1.661 ± 2.1580

2.384 ± 3.9340

0.006 ± 0.0110

5.333 ± 3.7870

0.000 ± 0.0000

3.902 ± 1.0920

2.617 ± 4.5160

1.096 ± 1.3840

1.046 ± 1.7870

1.446 ± 2.4620

0.166 ± 0.1650

5.791 ± 8.7620

1.090 ± 0.4010

4.789 ± 4.8820

0.103 ± 0.1780

0.383 ± 0.6630

0.356 ± 0.6170

0.290 ± 0.3520

0.348 ± 0.6030

0.075 ± 0.1290

1.538 ± 0.5840

0.280 ± 0.1430

0.945 ± 0.7320

5.454 ± 4.6270

1.068 ± 1.3700

0.000 ± 0.0000

0.896 ± 0.7890

1.413 ± 2.0800

23.101 ± 14.0690

0.352 ± 0.5930

0.236 ± 0.2120

0.006 ± 0.0050

0.041 ± 0.0720

4.231 ± 3.1140

12.882 ± 4.8250

0.364 ± 0.6230

0.141 ± 0.2450

0.116 ± 0.1850

1.026 ± 0.6100

0.597 ± 0.6500

1.737 ± 3.0010

5.425 ± 3.6020

0.000 ± 0.0000

13.448 ± 0.2930

Data are the mean percentage ± standard deviation of the mean; IPF, idiopathic pulmonary fibrosis; Other, includes patients with collagen vascular disease-associated interstitial lung disease.
