# Supplementary material for: Identification of Halophilic Microbes in Lung Fibrotic Tissue by Oligotyping
Source: Front Microbiol. 2018 Aug 30;9:1892. doi: 10.3389/fmicb.2018.01892 (PMC6127444; doi:10.3389/fmicb.2018.01892)
Supplement: Supplementary file 8 [file Table_4.DOC]

**Healthy subjects**

**(n=3)**

**Genera**

**IPF patients (n=3)**

**Supplementary Table 4. Genus frequency in BALF (%)**

**ance in BALF**

**Other**

**(n=2)**

*g__Actinobacillus*

*g__Actinomyces*

*g__Actinomycetaceae*

*g__Aerococcaceae*

*g__Aggregatibacter*

*g__Bacteroidales*

*g__Campylobacter*

*g__Capnocytophaga*

*g__Christensenellaceae*

*g__Clostridiaceae-1*

*g__Clostridiaceae-2*

*g__Clostridiales-1*

*g__Clostridium*

*g__Coprococcus*

*g__Fusobacterium*

*g__Gemellaceae*

*g__Granulicatella*

*g__Haemophilus*

*g__Halomonadaceae*

*g__Halomonas*

*g__Leptotrichia*

*g__Megasphaera*

*g__Neisseria*

*g__Neisseriaceae*

*g__Oribacterium*

*g__Pasteurellaceae*

*g__Pirellulaceae*

*g__Porphyromonas*

*g__Prevotella*

*g__Pseudomonas*

*g__Ruminococcaceae*

*g__Sediminibacterium*

*g__Selenomonas*

*g__Shewanella*

*g__SR1*

*g__Streptococcus*

*g__TM7-3*

*g__Veillonella*

0.000 ± 0.0000

1.998 ± 1.7400

4.142 ± 1.3320

0.000 ± 0.0000

0.314 ± 0.5450

1.271 ± 0.3220

0.656 ± 0.5700

0.171 ± 0.2960

0.000 ± 0.0000

0.085 ± 0.1480

0.342 ± 0.5920

0.256 ± 0.4440

0.513 ± 0.8880

0.000 ± 0.0000

0.342 ± 0.5920

0.000 ± 0.0000

0.171 ± 0.2960

0.171 ± 0.2960

14.375 ± 7.1420

0.844 ± 0.7980

1.469 ± 1.4140

1.671 ± 0.1890

1.929 ± 2.5060

0.171 ± 0.2960

0.000 ± 0.0000

0.000 ± 0.0000

0.085 ± 0.1480

1.015 ± 0.5410

11.907 ± 4.9990

15.229 ± 11.7190

1.282 ± 2.2210

2.997 ± 1.9700

0.342 ± 0.5920

1.756 ± 0.1530

0.000 ± 0.0000

8.894 ± 1.2120

0.000 ± 0.0000

5.040 ± 0.5210

0.000 ± 0.0000

0.607 ± 0.6880

0.693 ± 0.7660

0.000 ± 0.0000

0.000 ± 0.0000

0.291 ± 0.3380

0.191 ± 0.2500

0.183 ± 0.3110

0.043 ± 0.1060

0.007 ± 0.0180

0.043 ± 0.0720

0.164 ± 0.2270

0.535 ± 0.9510

0.111 ± 0.2440

0.134 ± 0.1780

0.212 ± 0.3470

0.191 ± 0.2110

8.400 ± 20.0280

41.508 ± 25.4290

0.000 ± 0.0000

0.071 ± 0.1140

0.171 ± 0.2650

0.331 ± 0.4720

0.000 ± 0.0000

0.057 ± 0.1390

0.000 ± 0.0000

0.000 ± 0.0000

0.219 ± 0.2770

3.542 ± 3.9860

8.066 ± 9.7670

0.051 ± 0.1240

4.470 ± 6.0350

0.704 ± 1.0000

18.230 ± 14.5060

0.125 ± 0.3070

2.172 ± 2.4660

0.000 ± 0.0000

1.224 ± 1.6700

0.000 ± 0.0000

2.486 ± 3.0010

1.562 ± 0.6670

0.000 ± 0.0000

0.133 ± 0.1880

3.472 ± 4.9100

0.924 ± 0.7920

0.643 ± 0.1190

0.000 ± 0.0000

0.000 ± 0.0000

0.000 ± 0.0000

0.283 ± 0.1150

0.009 ± 0.0130

0.000 ± 0.0000

0.206 ± 0.2910

0.238 ± 0.3370

0.814 ± 0.6370

0.476 ± 0.6740

16.014 ± 19.0070

0.000 ± 0.0000

0.394 ± 0.5570

1.208 ± 1.1940

1.361 ± 0.3820

0.064 ± 0.0910

0.435 ± 0.6150

0.000 ± 0.0000

0.000 ± 0.0000

0.147 ± 0.2070

15.383 ± 19.1840

13.718 ± 14.5410

0.000 ± 0.0000

6.589 ± 6.6240

2.161 ± 2.5410

5.444 ± 6.7010

0.005 ± 0.0060

5.952 ± 5.8460

0.000 ± 0.0000

5.216 ± 5.3200

Data are the mean percentage ± standard deviation of the mean; IPF, idiopathic pulmonary fibrosis;

Other, includes patients with collagen vascular disease-associated interstitial lung disease; BALF, bronchoalveolar lavage fluid.
