# Supplementary material for: Identification of Halophilic Microbes in Lung Fibrotic Tissue by Oligotyping
Source: Front Microbiol. 2018 Aug 30;9:1892. doi: 10.3389/fmicb.2018.01892 (PMC6127444; doi:10.3389/fmicb.2018.01892)
Supplement: Supplementary file 9 [file Table_5.DOC]

**Genera**

**Supplementary Table 5. Genus frequency in lung tissue (%)**

**IPF patients (n=3)**

**LC patients (n=3)**

**Other**

**(n=1)**

*g__Actinobacillus*

*g__Actinomyces*

*g__Actinomycetaceae*

*g__Aerococcaceae*

*g__Aggregatibacter*

*g__Bacteroidales*

*g__Campylobacter*

*g__Capnocytophaga*

*g__Christensenellaceae*

*g__Clostridiaceae-1*

*g__Clostridiaceae-2*

*g__Clostridiales-1*

*g__Clostridium*

*g__Coprococcus*

*g__Fusobacterium*

*g__Gemellaceae*

*g__Granulicatella*

*g__Haemophilus*

*g__Halomonadaceae*

*g__Halomonas*

*g__Leptotrichia*

*g__Megasphaera*

*g__Neisseria*

*g__Neisseriaceae*

*g__Oribacterium*

*g__Pasteurellaceae*

*g__Pirellulaceae*

*g__Porphyromonas*

*g__Prevotella*

*g__Pseudomonas*

*g__Ruminococcaceae*

*g__Sediminibacterium*

*g__Selenomonas*

*g__Shewanella*

*g__SR1*

*g__Streptococcus*

*g__TM7-3*

*g__Veillonella*

0.000 ± 0.0000

0.008 ± 0.0130

0.002 ± 0.0030

0.000 ± 0.0000

0.000 ± 0.0000

0.000 ± 0.0000

0.018 ± 0.0170

0.000 ± 0.0000

3.321 ± 0.7330

13.860 ± 0.5610

14.326 ± 1.0380

6.262 ± 0.6510

13.016 ± 1.5540

2.302 ± 0.2850

0.000 ± 0.0000

0.000 ± 0.0000

0.022 ± 0.0240

0.004 ± 0.0070

1.422 ± 0.7160

0.019 ± 0.0340

0.026 ± 0.0240

0.000 ± 0.0000

0.012 ± 0.0210

0.000 ± 0.0000

0.009 ± 0.0080

0.008 ± 0.0130

2.108 ± 0.3570

0.017 ± 0.0060

5.334 ± 0.1890

0.025 ± 0.0300

5.905 ± 0.2360

0.002 ± 0.0030

0.000 ± 0.0000

0.442 ± 0.2490

0.000 ± 0.0000

0.036 ± 0.0050

0.007 ± 0.0080

0.013 ± 0.0120

0.004 ± 0.0070

0.040 ± 0.0450

0.004 ± 0.0070

0.000 ± 0.0000

0.002 ± 0.0040

0.069 ± 0.1140

0.030 ± 0.0520

0.000 ± 0.0000

3.444 ± 0.2060

13.652 ± 0.2610

13.349 ± 2.1540

5.665 ± 0.4040

12.983 ± 2.6390

2.719 ± 0.2020

0.012 ± 0.0110

0.002 ± 0.0040

0.035 ± 0.0340

0.097 ± 0.1680

1.465 ± 0.5230

0.000 ± 0.0000

0.007 ± 0.0060

0.089 ± 0.1550

0.081 ± 0.1300

0.005 ± 0.0090

0.030 ± 0.0520

0.007 ± 0.0060

1.975 ± 0.1390

0.051 ± 0.0560

5.717 ± 2.3380

0.024 ± 0.0220

5.787 ± 0.6740

0.002 ± 0.0040

0.035 ± 0.0600

0.531 ± 0.2950

0.006 ± 0.0110

0.093 ± 0.0970

0.008 ± 0.0140

0.351 ± 0.5610

0.000

0.000

0.000

0.000

0.000

0.000

0.000

0.000

0.000

0.000

0.000

0.000

100.000

0.000

0.000

0.000

0.000

0.000

0.000

0.000

0.000

0.000

0.000

0.000

0.000

0.000

0.000

0.000

0.000

0.000

0.000

0.000

0.000

0.000

0.000

0.000

0.000

0.000

Data are the mean percentage ± standard deviation of the mean; IPF, idiopathic pulmonary fibrosis; LC, lung cancer.
