# Supplementary material for: Identification of Halophilic Microbes in Lung Fibrotic Tissue by Oligotyping
Source: Front Microbiol. 2018 Aug 30;9:1892. doi: 10.3389/fmicb.2018.01892 (PMC6127444; doi:10.3389/fmicb.2018.01892)
Supplement: Supplementary file 10 [file Table_6.DOCX]

**Supplementary Table 6. Results of PERMANOVA of phylogenetic distances among human samples (control, IPF, and lung cancer)**

Group 1 Group 2 Sample size Permutations pseudo-F p-value q-value

Healthy subjects IPF 14 999 2.249924349 0.089 0.267

Healthy subjects Lung cancer 9 999 1.986777319 0.071 0.267

Healthy subjects Other 5 999 0.371490504 0.903 0.903

IPF Lung cancer 17 999 1.1579364 0.299 0.4485

IPF Other 13 999 1.372115988 0.259 0.4485

Lung cancer Other 8 999 1.025896911 0.395 0.474

PERMANOVA, Permutational multivariate analysis of variance; IPF, idiopathic pulmonary fibrosis. Other refers to patients with collagen vascular disease-associated interstitial lung disease.
