# Supplementary material for: Identification of Halophilic Microbes in Lung Fibrotic Tissue by Oligotyping
Source: Front Microbiol. 2018 Aug 30;9:1892. doi: 10.3389/fmicb.2018.01892 (PMC6127444; doi:10.3389/fmicb.2018.01892)
Supplement: Supplementary file 11 [file Table_7.DOCX]

**Supplementary Table 7. Results of PERMANOVA of phylogenetic distances between murine groups**

**Group 1 Group 2 Sample size Permutations pseudo-F p-value q-value**

TGF-β1 TG WT 6 999 1.067676426 0.392 0.392

PERMANOVA, Permutational multivariate analysis of variance; TGF-β1, transforming growth factor-β1; TG, transgenic; WT, wild type.
