# Supplementary material for: Identification of Halophilic Microbes in Lung Fibrotic Tissue by Oligotyping
Source: Front Microbiol. 2018 Aug 30;9:1892. doi: 10.3389/fmicb.2018.01892 (PMC6127444; doi:10.3389/fmicb.2018.01892)
Supplement: Supplementary file 12 [file Table_8.DOCX]

**Supplementary Table 8. Results of PERMANOVA of phylogenetic distances of human samples by source (saliva, BALF, lung tissue)**

Group 1 Group 2 Sample size Permutations pseudo-F p-value q-value

BALF Lung tissue 10 999 9.522412509 0.005 0.0075

BALF Saliva 16 999 2.521960718 0.086 0.086

Lung tissue Saliva 18 999 13.58255594 0.001 0.003

PERMANOVA, Permutational multivariate analysis of variance; BALF, bronchoalveolar lavage fluid.
