# Supplementary material for: Identification of Halophilic Microbes in Lung Fibrotic Tissue by Oligotyping
Source: Front Microbiol. 2018 Aug 30;9:1892. doi: 10.3389/fmicb.2018.01892 (PMC6127444; doi:10.3389/fmicb.2018.01892)
Supplement: Supplementary file 13 [file Table_9.DOCX]

**Supplementary Table 9. Statistical differences in oligotype profiles between study groups by sample source**

**Taxon Sample source Group 1 Group 2 p-value**

*Christensenellaceae*

Lung tissue IPF LC 0.79

*Clostridium*

Saliva HS IPF 4.2e-05

Lung tissue IPF LC 0.91

*Shewanella*

Saliva HS IPF 0.28

BALF HS IPF 0.15

Lung tissue HS IPF 0.93

HS LC 0.90

IPF LC 0.81

*Halomonadaceae*

Saliva HS IPF 0.58

BALF HS IPF 0.18

Lung tissue IPF LC 0.82

LC, lung cancer; IPF, idiopathic pulmonary fibrosis; HS, healthy subjects; BALF, bronchoalveolar lavage fluid.
